# Quantitative Trait Loci Mapping of Mineral Element Contents in Brown Rice Using Backcross Inbred Lines Derived From *Oryza longistaminata*

**DOI:** 10.3389/fpls.2020.01229

**Published:** 2020-08-12

**Authors:** Xingdan Liu, Fengfeng Fan, Manman Liu, Weixiong Long, Yajie Yu, Huanran Yuan, Guojing Pan, Nengwu Li, Shaoqing Li, Jianfeng Liu

**Affiliations:** ^1^ College of Agronomy, Hunan Agricultural University, Changsha, China; ^2^ State Key Laboratory of Hybrid Rice, Key Laboratory for Research and Utilization of Heterosis in Indica Rice of Ministry of Agriculture, Engineering Research Center for Plant Biotechnology and Germplasm Utilization of Ministry of Education, College of Life Science, Wuhan University, Wuhan, China

**Keywords:** wild rice, *Oryza longistaminata*, mineral element, quantitative trait loci, backcross-inbred lines

## Abstract

Mineral elements play an extremely important role in human health, and are worthy of study in rice grain. Wild rice is an important gene pool for rice improvement including grain yield, disease, and pest resistance as well as mineral elements. In this study, we identified 33 quantitative trait loci (QTL) for Fe, Zn, Se, Cd, Hg, and As contents in wild rice *Oryza longistaminata*. Of which, 29 QTLs were the first report, and 12 QTLs were overlapped to form five clusters as *qSe1/qCd1* on chromosome 1, *qCd4.2/qHg4* on chromosome 4, *qFe5.2/qZn5.2* on chromosome 5, *qFe9/qHg9.2/qAs9.2* on chromosome 9, and *qCd10/qHg10* on chromosome 10. Importantly, *qSe1/qCd1*, can significantly improve the Se content while reduce the Cd content, and *qFe5.2/qZn5.2* can significantly improve both the Fe and Zn contents, they were delimited to an interval about 53.8 Kb and 26.2 Kb, respectively. These QTLs detected from *Oryza longistaminata* not only establish the basis for subsequent gene cloning to decipher the genetic mechanism of mineral element accumulation, but also provide new genetic resource for rice quality improvement.

## Introduction 

As one of the most important staple crops, rice provides more than 40% of the daily calories for the world’s population and is also an important source for protein, vitamins and minerals (Khush, 2001; Parengam et al., 2010). Mineral elements play an extremely important role on human health. However, micronutrient malnutrition, a major human health problem in the world, particularly among women and children in developing countries, is mainly resulted from a dietary deficiency in iron (Fe) and zinc (Zn) (Zeng et al., 2005; Liu et al., 2006). Micronutrient deficiencies can cause a range of problems, such as Fe deficiency leading to anaemia, Zn deficiency leading to stunted growth and intellectual disability, and selenium (Se) deficiency increasing the risk of cancer (Clark et al., 1996; Umeta et al., 2000; Finley et al., 2001). Therefore, increasing the content of mineral elements in rice has important nutritional value for people, especially for those who live rice as the staple food. However, as we know that not all mineral elements are beneficial to human health. Some heavy metals such as cadmium (Cd), mercury (Hg), and arsenic (As), pose a serious threat to human health (Takahashi et al., 2011; Zhang et al., 2014; Huang et al., 2015). Accumulation of the heavy metals in rice grains will seriously affect their edible value, thus, breeding rice varieties with high trace elements and less heavy metal are supposed to improve the health of people.

Molecular marker-assisted selection and genetic engineering are important and effective methods to improve rice quality (Masuda et al., 2013; Fan et al., 2017). Thus, exploring and identifying new genes or quantitative trait loci (QTL) for mineral element accumulation in *Oryza* species becomes the premise to breed mineral element-rich rice. For these reasons, more and more researches tend to explore genes or genetic loci for mineral element accumulation in rice. To date, there are 14 QTLs for Fe and Zn contents in unpolished rice grains having been identified by using RILs, and one of the main QTL was further analyzed to obtain ten candidate genes (Anuradha et al., 2012). Twenty QTLs for Fe, Zn, Se, Cd, and lead (Pb) content in brown rice have been identified from 378 accessions, and some of them are co-located, such as Cd and Pb on chromosome 5, Zn and Pb on chromosome 7, and Se and Pb on chromosome 11 (Huang et al., 2015). Moreover, some genes such as *OsNRAMP1* for Cd (Takahashi et al., 2011), *OsLCT1* for Cd (Uraguchi et al., 2011), *OsABCC1* for As (Song et al., 2014), and *OsHMA3* for Cd and Zn (Cai et al., 2019; Sui et al., 2019) have been cloned, they promote or repress mineral element accumulation through different pathways of mineral element uptake or transport, implying the complexity of genetic mechanism of mineral element accumulation in rice.

Wild rice, as an important gene pool for rice yield, quality, and resistance improvement, is also an essential genetic carrier for mineral element in nature (Londo et al., 2006; Garcia-Oliveira et al., 2009; Huang et al., 2013; Fan et al., 2019). Previous studies have identified some QTLs for mineral elements content in wild rice *Oryza rufipogon*, these QTLs have great potential in the subsequent gene cloning and breeding applications (Garcia-Oliveira et al., 2009). *Oryza longistaminata*, an ancient Africa wild species of AA genome, less genes have been explored for rice breeding apart from *Xa21* in the last 1990s. Here, we analyzed the QTLs for Fe, Zn, Se, Cd, Hg, and As contents using 127 backcross-inbred lines (BILs) derived from *O. longistaminata*. Totally, 40 QTLs for different minerals were identified, of which, 13 QTLs for trace elements Fe, Zn, and Se, and 16 QTLs for heavy metal elements Pb, Cd and As from *O. longistaminata* were the firstly reported, which will greatly help to breed high quality rice beneficial to human health-care.

## Materials and Methods

### Plant Materials and Field Planting

9311, an elite cultivated rice, was used as the recurrent parent for BIL population construction. *O. longistaminata*, a wild rice with a variety of excellent traits, was used as the donor parent. The population comprising 127 BILs was derived from a distant cross between *O. longistaminata* and 9311. Then, 9311 was used as the recurrent parent to backcross the hybrid 2 times, obtaining BC_2_F_1_. Finally, a set of 127 BC_2_F_16_ lines was constructed. The recurrent parent 9311 and 127 BILs were planted in the experimental field of Wuhan university experimental base in the summer of 2014 (Huashan, N30.54°, E114.52°) and 2015 (Ezhou, N30.40°, E114.88°), respectively. Each BIL and the recurrent parent consisted of 50 plants planted in five rows of 10 plants each adopting a uniform spacing of 20 cm between rows and 15 cm between plants. Three replications were performed by randomized complete block design.

### Analysis of Grain Element Content

After harvesting, rice grains were husked to obtain brown rice using five randomly-selected plants in each replicate. The unpolished grains were dried in hot air oven at 80°C for 24 h. Then ground to fine powder with a grinder, and filtered through a stainless-steel 0.3 mm mesh screen. To ensure consistency in elements analyses, seeds from every lot were analyzed as three replicates. Then 0.2 g was digested in 5 ml of HNO_3_–H_2_O_2_ (4:1 v/v) mixture. The Fe and Zn content in the digest solutions were determined with flame atomic absorption spectrometry (FLAA) (ContrAA 700, Analytik Jena AG, Germany). The Cd content in the digest solutions were determined with graphite furnace atomic absorption spectrometry (GFAA) (ContrAA 700, Analytik Jena AG, Germany). The Hg content in the digest solutions were determined with Atomic fluorescence spectrum (AFS) (PF6-2, Beijing General Analysis Instrument Co. LTD, China). Then 1.0 g was digested in 15 ml of HNO_3_–HClO_4_–HCl (10:1:4 v/v) mixture. The Se and As content in the digest solutions were determined with AFS.

### Date Analysis

Grain elements content were computed as parts per million (ppm). The statistical analyses and correlation analysis were performed with SPSS Statistics 20 (IBM, United States) using the mean values of each trait. Correlation among traits was computed at P < 0.05 and P < 0.01, respectively.

### QTL Analysis

The 127 BILs were subjected to whole-genome sequencing, and a high-quality bin map of ultrahigh-density SNPs was constructed based on the sequencing data (unpublished data). The DNA extraction, SNP screening, genotyping, bin map and genetic linkage map construction all adopt the previous reported methods (Jin et al., 2018; Fan et al., 2019). A total of 2,432 bins were obtained for the subsequent QTL analysis. The QTL mapping analysis was assembled from the genotype and phenotypic data using QTL IciMapping Version 3.3 software, and the interval mapping method of BIL module was selected (Meng et al., 2015). The logarithm of the odds (LOD) values (95% confidence interval) for the six traits were obtained through 1,000 permutations, and the average LOD threshold was 2.39. So the LOD score beyond 2.5 was defined as one QTL. The QTL were named as *qXY* (the X indicate the elements, the Y indicate the chromosome number).

### Gene and Protein Functional Domain Prediction

A putative gene in the QTL region was predicted by referring to the Rice Information GateWay (http://rice.hzau.edu.cn/rice/). The protein sequences of candidate genes were separately submitted to SMART (http://smart.embl-heidelberg.de/) to predict the functional domains using the domain annotation tool.

## Results

### Content of Mineral Elements in Brown Rice of BILs and Parent Line 9311

**Figure 1:**
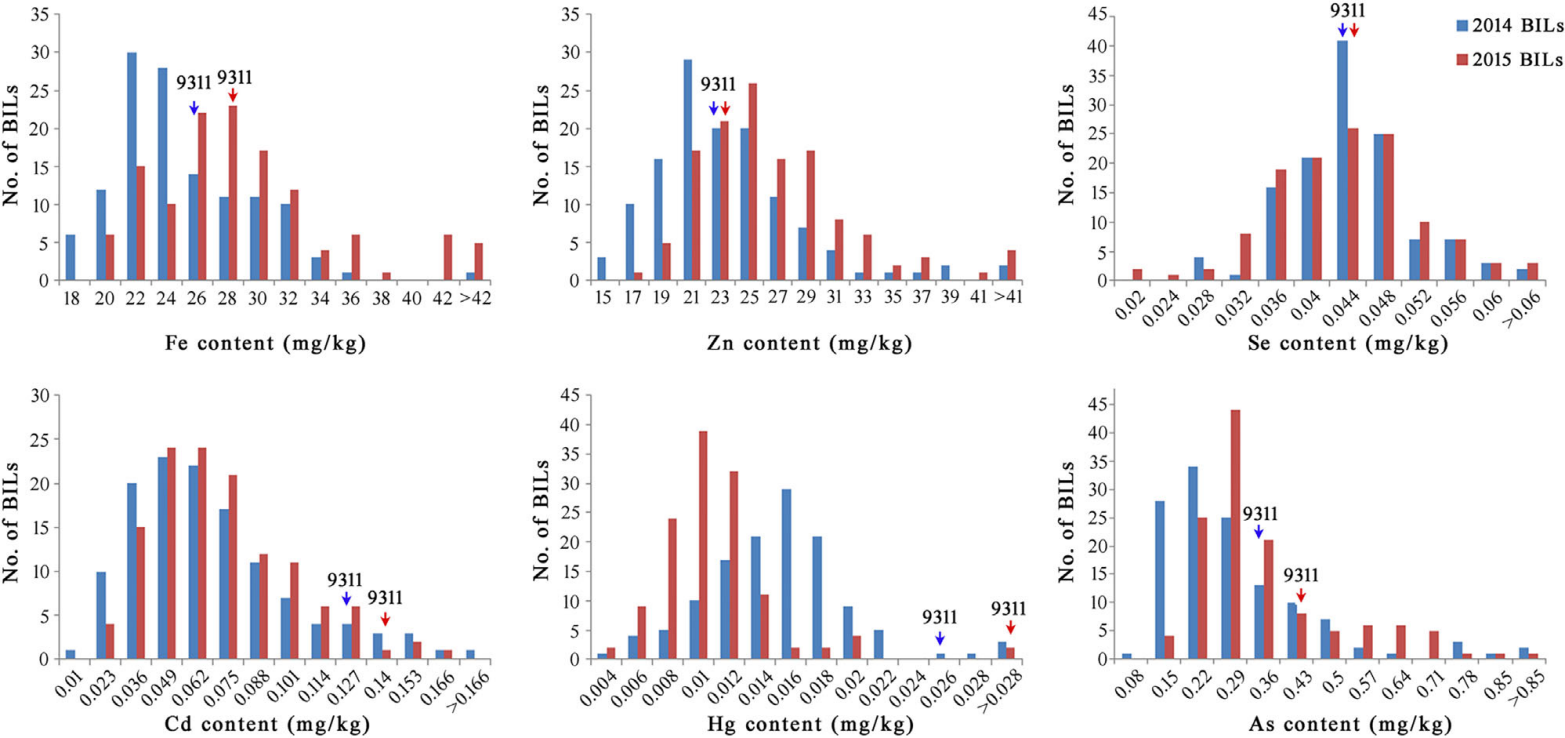
Distribution of six mineral element contents of BIL population in 2 years. Arrow head represents the content of 9311.

The mean, standard deviation (SD), coefficient of variation (CV), and range of the mineral elements for a total of 127 BILs were evaluated as presented in **Table 1**. Significant differences in mineral element contents were found. The means for trace elements such as Fe, Zn, and Se content were 24.10 mg/kg, 22.48 mg/kg and 0.043 mg/kg, respectively. The means for heavy metal elements such as Cd, Hg, and As content were 0.063 mg/kg, 0.015 mg/kg, and 0.266 mg/kg, respectively. At the same time, we also observed that the variation coefficient and range of each mineral element in the BILs were large, indicating a rich genetic diversity of the BILs (**Table 1**). Phenotypic values of the BILs were found being continuous and exhibited normal or skewed distribution patterns in all elements (**Figure 1**). It can be seen that the content of Fe, Zn, and As in the soil of Ezhou experimental base in 2015 was significantly higher than that of Huashan experimental base in 2014, while the content of Hg presented an opposite trend (**Table S1**). As a result, the content of Fe, Zn, and As in BIL group in 2015 was higher than that in 2014, while the content of Hg was lower than that in 2014. Although the contents of these mineral elements varied in different years, the overall trend remained consistent, which mean that this BIL population can be well used for the further QTL analysis.

**Table 1:** Phenotypic variation of the mineral elements in brown rice of 9311 and BIL lines in different environments.

Traits	9311	BILs (2014 Wuhan)			9311	BILs (2015 Wuhan)		
		Mean ± SD	CV%	Range		Mean ± SD	CV%	Range
Fe (mg/kg)	24.81	24.10 ± 4.80	19.92	15.13-51.32	26.82	27.99 ± 6.46	23.08	18.02-55.41
Zn (mg/kg)	21.93	22.48 ± 5.15	22.91	12.69-42.49	21.29	25.62 ± 5.54	21.62	16.20-47.23
Se (mg/kg)	0.044	0.043 ± 0.008	18.60	0.025-0.083	0.041	0.042 ± 0.009	21.43	0.009-0.066
Cd (mg/kg)	0.127	0.063 ± 0.036	57.14	0.010-0.237	0.129	0.065 ± 0.030	46.15	0.016-0.158
Hg (mg/kg)	0.035	0.015 ± 0.012	80.00	0.004-0.143	0.025	0.010 ± 0.007	70.00	0.003-0.074
As (mg/kg)	0.422	0.266 ± 0.177	66.54	0.079-1.251	0.329	0.324 ± 0.160	49.38	0.130-0.970

### Correlation Among Six Mineral Elements

In order to understand the potential relationships between the six mineral elements, correlation analysis was performed for the mineral elements in the 127 BILs (**Table 2**). Results showed that a significantly positive correlation was detected between Fe and Zn in 2 years, and an extremely significant correlation was observed between Zn and Se in both 2014 and 2015. The other elements showed a weak correlation with each other.

**Table 2:** Correlation coefficients among elements of BILs in 2014 (upper right) and 2015 (lower left).

Traits	Fe	Zn	Se	Cd	Hg	As
**Fe**	1	0.222**	0.111	-0.149*	-0.019	0.016
**Zn**	0.399**	1	0.235**	0.114	0.157*	0.026
**Se**	0.164*	0.374**	1	-0.111	0.124	-0.093
**Cd**	0.026	0.076	-0.028	1	0.108	-0.029
**Hg**	0.146	0.177*	0.143	0.004	1	-0.062
**As**	0.059	-0.138	-0.076	0.046	-0.075	1

*Correlation significant at the 0.05 level. **Correlation significant at the 0.01 level.

### QTL Mapping for Mineral Elements

**Figure 2:**
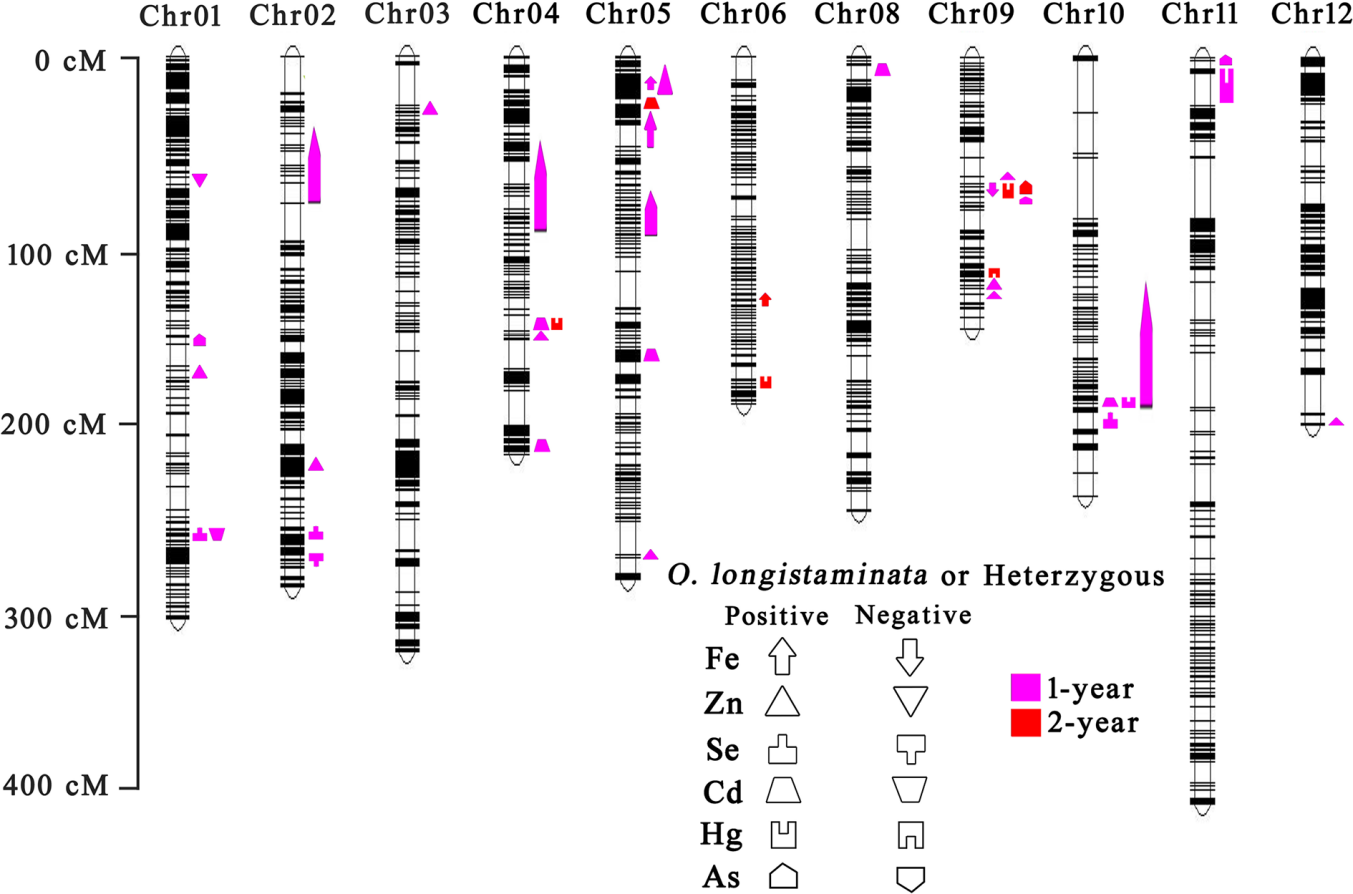
QTL analysis of six mineral element contents of BIL population in different years. Left is the scale for the genetic length of each chromosome. The upward direction indicates the *O. longistaminata* parental allele or heterozygous genotype for each locus showing positive effects. The downward direction indicates the *O. longistaminata* allele or heterozygous genotype for each locus showing negative effects. The pink indicates QTLs detected in 1 year. The red indicates QTLs detected in 2 years.

Interval mapping revealed that a total of 40 QTL for six mineral element contents were identified on all chromosomes except chromosome 7 (**Figure 2**). Of which, 15 QTL for trace element contents and 18 QTL for heavy metal element contents were derived from wild rice *O. longistaminata* (**Table S2**).

For trace elements, a total of 19 QTLs were detected in 2 years (**Figure 2**). Among these, 15 QTLs with positive effects were detected from wild rice *O. longistaminata* (**Table S2**). In details, three QTLs for Fe content were mapped on chromosomes 5, 5 and 6, and explained 6.8%, 10.0%, and 32.4% of the phenotypic variation, respectively. Ten QTLs for Zn content explained the phenotypic variation ranging from 4.7% to 16.6%. Two QTLs for Se content were mapped on chromosomes 1 and 2, and explained about 10.3% and 13.9% of the variance, respectively (**Table S2**). As for heavy metal elements, 21 QTLs were identified in 2 years, and 18 QTLs with positive effects were detected from *O. longistaminata* (**Figure 2** and **Table S2**). Of which, five QTLs for Cd content were mapped on chromosomes 4, 4, 5, 5, and 10, and explained 8.4%, 25.1%, 21.0%, 33.4%, and 23.9% of the phenotypic variations, respectively. Five QTLs, *qHg4*, *qHg6*, *qHg9.2*, *qHg10*, and *qHg11* were detected for Hg content, and explained the phenotypic variations ranging from 15.0% to 27.7%. Eight QTLs were detected for As content and explained 25.6%~54.6% of the phenotypic variation (**Table S2**).

Notably, the *qFe6* for Fe content, *qZn4* for Zn content, *qCd5.2* for Cd content, *qAs9.2* for As content and *qHg4*, *qHg6*, *qHg9.2* for Hg content were repeatedly identified from *O. longistaminata* in 2 years, meaning their potentially great value in rice breeding practice.

### QTL Co-Localization for Different Elements

Co-localization of QTL for different element content in grains is common in rice (Garcia-Oliveira et al., 2009; Zhang et al., 2014; Huang et al., 2015). In our study, five cases of QTL colocalization, i.e. Se and Cd on chromosome 1, Cd and Hg on chromosome 4, Fe, and Zn on chromosome 5, Fe, Hg, and As on chromosome 9 and Cd, Hg on chromosome 10, were observed (**Figure 2**). Among them, the *qSe1/qCd1* site explained 10.3% of the phenotypic variance for Se content and 6.9% for Cd content in the BIL population. The *qFe5.2*/*qZn5.2* site explained 10.0% of the phenotypic variance for Fe content and 13.2% for Zn content. The *qCd4.2/qHg4*, *qFe9/qHg9.2/qAs9.2* and *qCd10/qHg10* site explained the phenotypic variation ranging from 12.8% to 29.2%.

### Validation of qSe1/qCd1 and qFe5.2/qZn5.2

**Figure 3:**
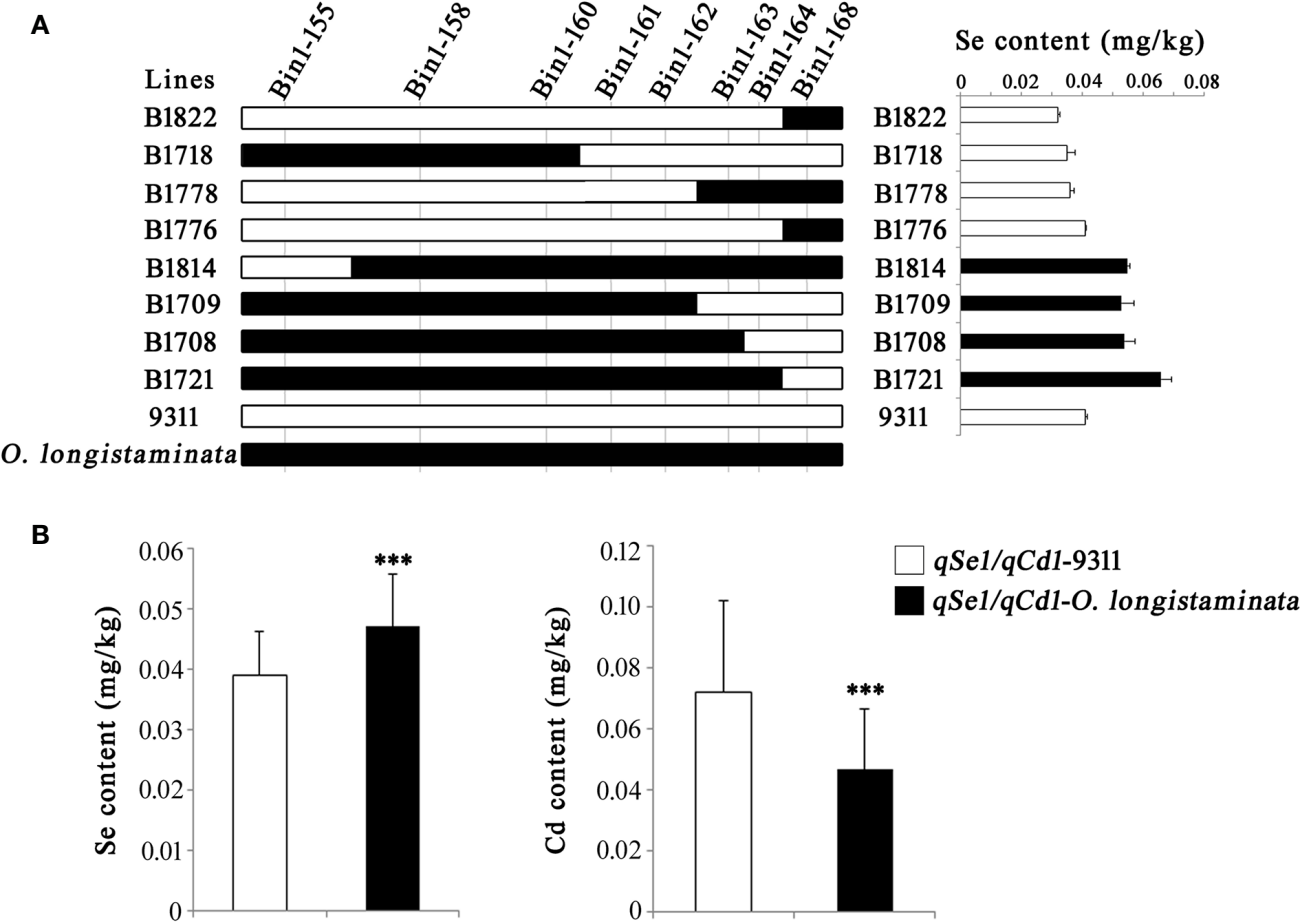
Validation of the function of *qSe1/qCd1*. **(A)** Progeny testing. **(B)** Analysis of the Se and Cd content of the BIL population. ***p < 0.001.

**Figure 4:**
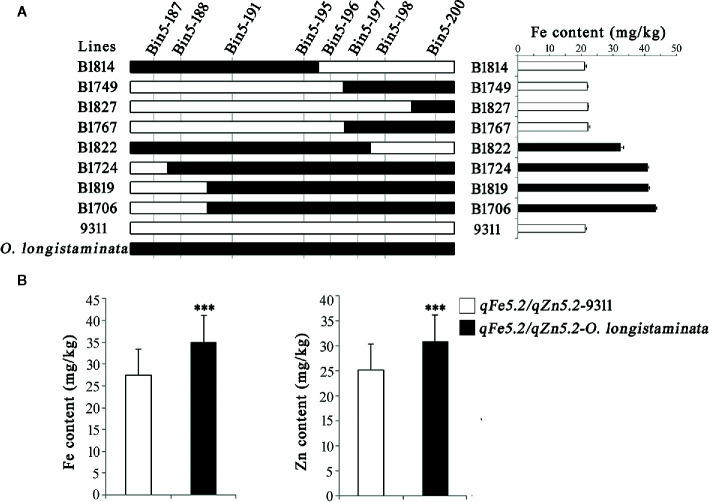
Validation of the function of *qFe5.2*/*qZn5.2*. **(A)** Progeny testing. **(B)** Analysis of the Fe and Zn content of the BIL population. ***p < 0.001.

In order to further validate the functions of the newly detected QTLs, we analyzed the genetic effects of the *qSe1/qCd1* and *qFe5.2*/*qZn5.2*. Results showed that the Bin1-161 and Bin1-162 well defines the core interval of *qSe1/qCd1* contributing to the Se and Cd contents in the BIL lines (**Figure 3**), and the Se and Cd content of the lines containing *qSe1/qCd1* from *O. longistaminata* were about 21% higher and 35% lower than those lines with 9311 fragments, respectively (**Figure 3B**). The other site, *qFe5.2*/*qZn5.2*, was delimited by the tightly linked marker Bin5-196 (**Figure 4A**). Surprisingly, the Fe and Zn content in the lines with *qFe5.2*/*qZn5.2* were about 28% and 23% higher than those no *qFe5.2*/*qZn5.2* lines, respectively (**Figure 4B**). These results indicate that *qSe1/qCd1* and *qFe5.2*/*qZn5.2* from *O. longistaminata* could significantly improve the nutritional value of mineral elements in rice.

## Discussion

Although rice is not considered as a main source of mineral elements, this does not exclude the importance to breed novel rice varieties with high content of the trace elements including Fe, Zn, and Se (Zhang et al., 2014; Huang et al., 2015). Recently, more and more attention has been paid to the research of Zn- and Se-rich rice, because of a variety of health-care functions of both Zn and Se on human health (Liu et al., 2015; Xu et al., 2016; Yuan et al., 2017; D’Amato et al., 2018). On the other hand, with the aggravation of the industrialization process, the enrichment of heavy metals in rice has become increasingly prominent, especially the enrichment of Cd, which has seriously threatened the health of people living with rice (Takahashi et al., 2011; Ueno et al., 2011). Therefore, breeding of rice varieties with high trace elements and low heavy metals, especially those rich in Zn/Se and low in Cd, is becoming the aim of breeders.

In order to achieve this goal, predecessors have done a lot of preliminary work. Some QTLs related to mineral element accumulation identified from cultivated rice and common wild rice have been successfully applied in production (Garcia-Oliveira et al., 2009; Nawaz et al., 2015). In this study, we focused on wild rice *O. longistaminata*, an Africa-derived important genetic resource that less favorable genes were explored (Zhang et al., 2015; Fan et al., 2017; Jin et al., 2018; Fan et al., 2019). Here, we firstly identified 33 QTLs for content of six mineral elements from *O. longistaminata*. Apart from the the *qFe5.1*, *qFe6*, *qAs4*, and *qAs10* were overlapped with the Zinc-iron transporter gene *OsZIP7a*, *OsZIP12* (Yang et al., 2009; Liu et al., 2019), and the arsenate reductase gene *OsHAC1.2* and *OsACR2.1* (Duan et al., 2007; Shi et al., 2016), respectively (**Table S2**), the other 29 QTLs found in *O. longistaminata* are different from the others. These results indicate that *O. longistaminata* contains abundant genetic resources for mineral element content improvement.

In addition, we also found that these QTLs tend to act in cluster. Among them, *qSe1/qCd1*, a QTL cluster improve the Se and Cd contents in the BILs, was delimited to an interval about 53.8 Kb on chromosome 1 (**Figure 3**). This locus contains three hypothetical protein and seven function genes including *MH01g0727800* (**Table 3**). The *MH01g0727800* encodes a CHD3-type chromatin-remodeling factor PICKLE, which is belonging to the CHD3 family (Hu et al., 2013). CHD3 protein can interact with H3K4me2 and H3K27me3 by the chromodomains and plant homeodomain finger, respectively (Hu et al., 2012). Many studied have demonstrated that histone methylation modification plays numerous important roles in the growth and development of rice (Cui et al., 2013; Li et al., 2013; Yan et al., 2015). Therefore, we speculate that this gene may play an important role in regulating the Se and Cd contents. Another QTL cluster, *qFe5.2*/*qZn5.2*, which can significantly improve the content of both Fe and Zn in rice grains, was located in an interval about 26.2 Kb on chromosome 5 (**Figure 4**). This locus containing only four predicted function genes (**Table 3**). Of which, *MH05g0033700* encoding a phosphatidylinositol-specific phospholipase C, which effectively influences the metal ion signal transduction (Petersen and Wakui, 1990; Meldrum et al., 1991). *MH05g0033800*, containing a serine/threonine protein kinases domain and plays an important role in metal ion transport (Johnson and Wu, 2016). The discovery will help us to further understand the molecular mechanisms of mineral element accumulation in rice seeds.

**Table 3:** The predicted functional genes at loci of *qSe1/qCd1* and *qFe5.2/qZn5.2*.

Loci	Gene names	Gene function
***qSe1/qCd1***	MH01g0727000	Hypothetical protein
	MH01g0727100	glycosyl transferase, putative, expressed
	MH01g0727200	Pectinesterase, putative, expressed
	MH01g0727300	Powdery mildew resistant protein 5, putative, expressed
	MH01g0727400	Hypothetical protein
	MH01g0727500	Hypothetical protein
	MH01g0727600	Acyl-desaturase, chloroplast precursor, putative, expressed
	MH01g0727700	Pentatricopeptide, putative, expressed
	MH01g0727800	CHD3-type chromatin-remodeling factor PICKLE.
	MH01g0727900	ANTH domain containing protein, expressed
***qFe5.2/qZn5.2***	MH05g0033700	phospholipase C, putative, expressed
	MH05g0033800	TKL_IRAK_CR4L.4—The CR4L subfamily has homology with Crinkly4, expressed
	MH05g0033900	dnaJ domain containing protein, expressed
	MH05g0034000	flavonol synthase/flavanone 3-hydroxylase, putative, expressed

**Figure 5:**
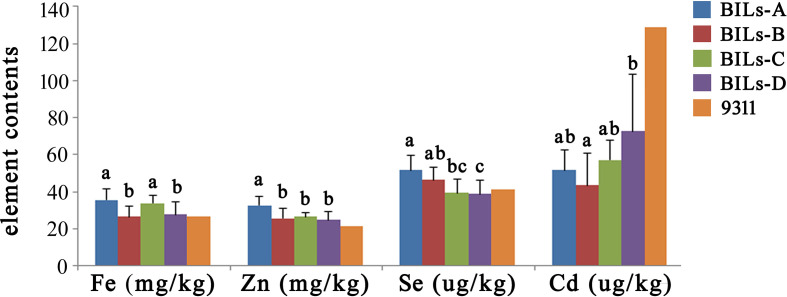
Genotype analysis and element content performance of different subpopulations. BILs-A containing both of *qSe1/qCd1* and *qFe5.2/qZn5.2*, BILs-B containing only *qSe1/qCd1*, BILs-C containing only *qFe5.2/qZn5.2*, BILs-D containing no any of these favorable QTL clusters. 9311 was used as a control. The letters at the top of bars represent significant differences (P < 0.05) as determined by Student’s t test.

Pyramiding multiple favorable genes in a single material is considered to be an important method of rice breeding (Brunner et al., 2010; Fan et al., 2017; Zeng et al., 2017). In order to further study the application value of these new QTLs, we conducted a comprehensive analysis of the BILs. Firstly, we divided the BIL population into four different subpopulations: BILs-A containing both of *qSe1/qCd1* and *qFe5.2/qZn5.2*, BILs-B containing only *qSe1/qCd1*, BILs-C containing only *qFe5.2/qZn5.2* and BILs-D containing no any of these favorable QTL clusters. We further analyzed the Fe, Zn, Se, and Cd content of different groups. Results showed that the Fe, Zn and Se content of BILs-A were higher than other groups, while the Cd content was lower than other groups (**Figure 5**). These results indicated that the BILs pyramided with *qSe1/qCd1* and *qFe5.2*/*qZn5.2* exhibit an ideal state of high trace elements and low heavy metals, which showed great application potential in rice nutritional quality improvement.

## Conclusions

We dissected the genetic basis for mineral element contents of Fe, Zn, and Se, and heavy mental contents of Cd, Pb, and As in African wild rice *Oryza longistaminata* and identified 29 novel QTL for these traits. Among the new QTLs, *qSe1/qCd1* and *qFe5.2*/*qZn5.2* largely contributed to Fe, Zn, Se, and Cd contents in BILs, and provide a basis for subsequent breeding applications and gene cloning. This work provided new ideas to explain the genetic mechanism of mineral element accumulation

## Data Availability Statement

All datasets generated for this study are included in the article/**Supplementary Material.**

## Author Contributions

JL, SL, and FF designed the research. XL, FF, ML, WL, HY, GP, and NL performed field experiment and QTL analysis. XL, FF, and SL analyzed data and wrote the manuscript. All authors contributed to the article and approved the submitted version.

## Conflict of Interest

The authors declare that the research was conducted in the absence of any commercial or financial relationships that could be construed as a potential conflict of interest.
