# Supplementary material for: Quantitative Trait Loci Mapping of Mineral Element Contents in Brown Rice Using Backcross Inbred Lines Derived From Oryza longistaminata
Source: Front Plant Sci. 2020 Aug 12;11:1229. doi: 10.3389/fpls.2020.01229 (PMC7434966; doi:10.3389/fpls.2020.01229)
Supplement: Supplementary file 1 [file Table_1.docx]

**QTL mapping of mineral element contents in brown rice using backcross inbred lines derived from *Oryza longistaminata***

Xingdan Liu^1†^, Fengfeng Fan^2†^, Manman Liu^2^, Weixiong Long^2^, Yajie Yu^2^, Huanran Yuan^2^, Guojing Pan^2^, Nengwu Li^2^, Shaoqing Li^2^*, Jianfeng Liu^1^*

^1^College of Agronomy, Hunan Agricultural University, Changsha 410128, China

^2^State Key Laboratory of Hybrid Rice, Key Laboratory for Research and Utilization of Heterosis in Indica Rice of Ministry of Agriculture, Engineering Research Center for Plant Biotechnology and Germplasm Utilization of Ministry of Education, College of Life Science, Wuhan University, Wuhan 430072, China.

*Corresponding author:

Jianfeng Liu

Email: liujianfeng@hunau.edu.cn

Shaoqing Li

Email: shaoqingli@whu.edu.cn

^†^These authors have contributed equally to this work

**Table S1** The contents of Fe, Zn, Se, Cd, Hg and As in the experimental fields in different regions

| **Regions** | **Fe**(mg/kg) | **Zn**(mg/kg) | **Se**(mg/kg) | **Cd**(mg/kg) | **Hg**(mg/kg) | **As**(mg/kg) |
| --- | --- | --- | --- | --- | --- | --- |
| **2014, Huanshan** | 26300±1088 | 53.19±4.70 | 0.306±0.006 | 0.225±0.075 | 0.272±0.007 | 3.007±0.091 |
| **2015, Ezhou** | 30963±1184 | 88.69±8.05 | 0.441±0.013 | 0.161±0.019 | 0.150±0.002 | 5.007±0.990 |

**Table S2** QTL information for concentrations of Fe, Zn, Se, Cd, Hg and As of brown rice in BILs

| Triats | QTLs | Population | Chr. | L/Bin | R/Bin | L/bp | R/bp | LOD | Add | PVE (%) | Reported |
| --- | --- | --- | --- | --- | --- | --- | --- | --- | --- | --- | --- |
| Fe | *qFe5.1* | 2014 BILs | 5 | 5-182 | 5-183 | 5772409_6032310 | 3752165_3767025 | 2.7 | 2.0 | 6.8 | *OsZIP7a* |
|  | *qFe5.2* | 2015 BILs | 5 | 5-196 | 5-197 | 1395818_1422012 | 1422356_1465849 | 3.3 | 3.1 | 10.0 |  |
|  | *qFe6* | 2014 BILs | 6 | 6-84 | 6-85 | 20497138_20686584 | 20686585_20881453 | 11.3 | 10.9 | 32.4 | *OsZIP12* |
|  |  | 2015 BILs | 6 | 6-84 | 6-85 | 20497138_20686584 | 20686585_20881453 | 4.6 | 9.3 | 13.4 |  |
|  | *qFe9* | 2014 BILs | 9 | 9-54 | 9-55 | 10659894_10747290 | 11744813_12091346 | 4.6 | -3.8 | 12.8 |  |
| Zn | *qZn1.1* | 2014 BILs | 1 | 1-11 | 1-12 | 9338700_9355837 | 9355838_9606753 | 3.7 | -1.6 | 7.4 |  |
|  | *qZn1.2* | 2014 BILs | 1 | 1-320 | 1-321 | 22786059_22833177 | 22680874_22783937 | 6.6 | 2.1 | 13.9 |  |
|  | *qZn2* | 2015 BILs | 2 | 2-75 | 2-76 | 27233655_27493778 | 27221931_27233070 | 2.9 | 1.4 | 4.7 |  |
|  | *qZn3* | 2014 BILs | 3 | 3-85 | 3-86 | 2402978_2453420 | 2205060_2402604 | 3.9 | 4.7 | 16.6 |  |
|  | *qZn4* | 2014 BILs | 4 | 4-70 | 4-71 | 23897139_23936438 | 23855540_23896420 | 2.6 | 5.2 | 13.0 |  |
|  |  | 2015 BILs | 4 | 4-71 | 4-72 | 23855540_23896420 | 22995875_23068116 | 4.7 | 8.9 | 8.2 |  |
|  | *qZn5.1* | 2015 BILs | 5 | 5-37 | 5-38 | 26512788_26529692 | 26281199_26512787 | 3.0 | 1.4 | 8.9 |  |
|  | *qZn5.2* | 2015 BILs | 5 | 5-207 | 5-208 | 2275757_2327181 | 2327248_236614 | 5.2 | 2.1 | 13.2 |  |
|  | *qZn9.1* | 2014 BILs | 9 | 9-16 | 9-17 | 19779493_19925189 | 19674399_19779476 | 5.6 | 4.3 | 13.7 |  |
|  | *qZn9.2* | 2015 BILs | 9 | 9-26 | 9-27 | 18115769_18320575 | 17858000_18115768 | 6.3 | 5.5 | 15.9 |  |
|  | *qZn9.3* | 2015 BILs | 9 | 9-65 | 9-66 | 9555157_9639316 | 9514857_9550161 | 3.3 | 8.5 | 7.4 |  |
|  | *qZn12* | 2014 BILs | 12 | 12-184 | 12-185 | 24539163_24556330 | 24275509_24531435 | 3.4 | 1.7 | 7.0 |  |
| Se | *qSe1* | 2015 BILs | 1 | 1-161 | 1-162 | 36539238_36569505 | 36515725_36536296 | 3.8 | 0.003 | 10.3 |  |
|  | *qSe2.1* | 2015 BILs | 2 | 2-10 | 2-11 | 33732145_33904074 | 33476295_33732144 | 2.6 | -0.003 | 6.9 |  |
|  | *qSe2.2* | 2015 BILs | 2 | 2-36 | 2-37 | 31140724_31253649 | 31034261_31140723 | 5.1 | 0.005 | 13.9 |  |
|  | *qSe10* | 2014 BILs | 10 | 10-121 | 10-122 | 20723576_20782580 | 22846234_22859183 | 3.1 | -0.007 | 9.2 | *qSe10* |
| Cd | *qCd1* | 2015 BILs | 1 | 1-161 | 1-162 | 36539238_36569505 | 36515725_36536296 | 8.4 | -0.02 | 6.9 |  |
|  | *qCd4.1* | 2015 BILs | 4 | 4-2 | 4-3 | 32025274_32054678 | 32000283_32022813 | 3.1 | 0.01 | 8.4 |  |
|  | *qCd4.2* | 2014 BILs | 4 | 4-74 | 4-75 | 23506168_23854299 | 22956798_22995874 | 4.3 | 0.05 | 25.1 |  |
|  | *qCd5.1* | 2014 BILs | 5 | 5-128 | 5-129 | 17346596_17378319 | 17378829_17451085 | 3.2 | 0.07 | 21.0 |  |
|  | *qCd5.2* | 2014 BILs | 5 | 5-184 | 5-185 | 3736831_3752164 | 3767026_3794683 | 3.0 | 0.04 | 33.4 |  |
|  |  | 2015 BILs | 5 | 5-184 | 5-185 | 3736831_3752164 | 3767026_3794683 | 2.6 | 0.02 | 14.1 |  |
|  | *qCd8* | 2015 BILs | 8 | 8-110 | 8-111 | 1925603_1983883 | 1902547_1925303 | 2.6 | -0.01 | 6.9 |  |
|  | *qCd10* | 2014 BILs | 10 | 10-109 | 10-110 | 19619797_19942850 | 19944071_19995911 | 4.1 | 0.06 | 23.9 |  |
| Hg | *qHg4* | 2014 BILs | 4 | 4-74 | 4-75 | 23506168_23854299 | 22956798_22995874 | 12.2 | 0.02 | 25.1 |  |
|  |  | 2015 BILs | 4 | 4-74 | 4-75 | 23506168_23854299 | 22956798_22995874 | 8.2 | 0.01 | 20.3 |  |
|  | *qHg6* | 2014 BILs | 6 | 6-16 | 6-17 | 27405883_27514883 | 27339655_27405882 | 6.2 | 0.01 | 15.0 |  |
|  |  | 2015 BILs | 6 | 6-15 | 6-16 | 27514884_27547936 | 27405883_27514883 | 6.7 | 0.01 | 16.8 |  |
|  | *qHg9.1* | 2014 BILs | 9 | 9-28 | 9-29 | 17842644_17857999 | 16687703_17842643 | 8.1 | -0.01 | 20.5 |  |
|  |  | 2015 BILs | 9 | 9-28 | 9-29 | 17842644_17857999 | 16687703_17842643 | 7.6 | -0.01 | 19.4 |  |
|  | *qHg9.2* | 2014 BILs | 9 | 9-52 | 9-53 | 12477001_12513874 | 10645237_10659893 | 15.3 | 0.04 | 27.7 |  |
|  |  | 2015 BILs | 9 | 9-52 | 9-53 | 12477001_12513874 | 10645237_10659893 | 10.4 | 0.02 | 22.2 |  |
|  | *qHg10* | 2015 BILs | 10 | 10-109 | 10-110 | 19619797_19942850 | 19944071_19995911 | 9.1 | 0.02 | 22.2 |  |
|  | *qHg11* | 2014 BILs | 11 | 11-110 | 11-111 | 4141694_4170138 | 1337407_1355746 | 9.7 | 0.04 | 27.7 |  |
| As | *qAs1* | 2014 BILs | 1 | 1-78 | 1-79 | 20867480_20898504 | 20899281_20918687 | 4.6 | 0.3 | 54.6 |  |
|  | *qAs2* | 2014 BILs | 2 | 2-248 | 2-249 | 3713059_3993375 | 10416512_10475929 | 3.6 | 0.3 | 51.6 |  |
|  | *qAs4* | 2014 BILs | 4 | 4-123 | 4-124 | 15139044_15284012 | 6561131_8468212 | 3.0 | 0.3 | 44.8 | *OsHAC1.2* |
|  | *qAs5* | 2014 BILs | 5 | 5-135 | 5-136 | 7454274_7471652 | 10400415_10427983 | 3.8 | 0.3 | 44.5 |  |
|  | *qAs9.1* | 2014 BILs | 9 | 9-58 | 9-59 | 12222432_12476174 | 12515610_12531168 | 3.5 | 0.3 | 27.7 |  |
|  | *qAs9.2* | 2014 BILs | 9 | 9-61 | 9-62 | 10748697_11114176 | 9803582_9840859 | 3.3 | 0.2 | 25.6 |  |
|  |  | 2015 BILs | 9 | 9-60 | 9-61 | 11114177_11721737 | 10748697_11114176 | 3.3 | 0.2 | 29.2 |  |
|  | *qAs10* | 2015 BILs | 10 | 10-9 | 10-10 | 21626668_21647048 | 12626156_12698496 | 5.6 | 0.2 | 69.7 | *OsACR2.1* |
|  | *qAs11.1* | 2014 BILs | 11 | 11-127 | 11-128 | 370956_395333 | 1_370955 | 4.4 | 0.3 | 54.4 |  |

Add: additive effect; positive number represents the positive effect fragment from *O. Longistaminata*; negative number represents the positive effect fragment from 9311.
